# Modelling the spread and control of *Xylella fastidiosa* in the early stages of invasion in Apulia, Italy

**DOI:** 10.1007/s10530-017-1393-5

**Published:** 2017-02-21

**Authors:** Steven M. White, James M. Bullock, Danny A. P. Hooftman, Daniel S. Chapman

**Affiliations:** 1 Centre for Ecology & Hydrology, Benson Lane, Crowmarsh Gifford, Wallingford, Oxfordshire OX10 8BB UK; 2 Mathematical Institute, University of Oxford, Andrew Wiles Building, Radcliffe Observatory Quarter, Woodstock Road, Oxford, Oxfordshire OX2 6GG UK; 3 Lactuca: Environmental Data Analyses and Modelling, 1112 NC Diemen, The Netherlands; 4 Centre for Ecology & Hydrology, Bush Estate, Penicuik, Midlothian EH26 0QB UK

**Keywords:** Buffer zone, CoDiRO, *Olea europaea*, Olive quick decline syndrome, Pierce’s disease, *X. fastidiosa*

## Abstract

*Xylella fastidiosa* is an important plant pathogen that attacks several plants of economic importance. Once restricted to the Americas, the bacterium, which causes olive quick decline syndrome, was discovered near Lecce, Italy in 2013. Since the initial outbreak, it has invaded 23,000 ha of olives in the Apulian Region, southern Italy, and is of great concern throughout Mediterranean basin. Therefore, predicting its spread and estimating the efficacy of control are of utmost importance. As data on this invasive infectious disease are poor, we have developed a spatially-explicit simulation model for *X. fastidiosa* to provide guidance for predicting spread in the early stages of invasion and inform management strategies. The model qualitatively and quantitatively predicts the patterns of spread. We model control zones currently employed in Apulia, showing that increasing buffer widths decrease infection risk beyond the control zone, but this may not halt the spread completely due to stochastic long-distance jumps caused by vector dispersal. Therefore, management practices should aim to reduce vector long-distance dispersal. We find optimal control scenarios that minimise control effort while reducing *X. fastidiosa* spread maximally—suggesting that increasing buffer zone widths should be favoured over surveillance efforts as control budgets increase. Our model highlights the importance of non-olive hosts which increase the spread rate of the disease and may lead to an order of magnitude increase in risk. Many aspects of *X. fastidiosa* disease invasion remain uncertain and hinder forecasting; we recommend future studies investigating quantification of the infection growth rate, and short and long distance dispersal.

**Electronic supplementary material:**

The online version of this article (doi:10.1007/s10530-017-1393-5) contains supplementary material, which is available to authorized users.

## Introduction

The magnitude of negative impacts on the economy, native biota and human society caused by non-native invasive species is increasing rapidly (Pimentel et al. 2005; Simberloff et al. 2013). This fact holds for emerging infectious diseases (EIDs) of plants, and the prevalence of invasive plant diseases is increasing due to trade and transport globalization (Hulme 2009; Dehnen-Schmutz et al. 2010), as well as climate change (Gautam et al. 2013). There is a clear need to develop strategies to manage the emergence, spread and impacts of these diseases (Baker and Bode 2016), but for many EIDs, novel environments or a general lack of data make predicting future distributions or rates of spread difficult. Despite this, modelling efforts can help to understand better the spread of new diseases as well as provide testable theory and guidance on effective control strategies. For example, Richter et al. (2013) use a spread model to show that an optimally-designed management plan consisting of survey and eradication can drastically reduce the spread of allergenic ragweed, *Ambrosia artemisiifolia*, resulting in substantial saving in medical costs. Parnell et al. (2015) use a simple spread model to reveal a rule of thumb for early detection surveillance strategies for EIDs of plants. However, it is rare to use spread models in plant health risk assessment in contrast to non-mechanistic species distribution models (Chapman et al. 2015).

Here we derive and analyse a novel spread model to investigate control of an emerging outbreak of *Xylella fastidiosa* in Italy (Martelli et al. 2015), modelling a buffer zone management strategy. *X. fastidiosa* is a xylem-limited Gram-negative bacterium and the recognised agent of a number of severe and economically-important diseases, including Pierce’s disease of grapevines, citrus variegated chlorosis (CVC), and other disorders of perennial crops and landscape plants (Purcell and Hopkins 1996). Once restricted to the Americas, a new invasive strain, known as CoDiRO (Saponari et al. 2013), was discovered near Lecce, Italy in October 2013 (Loconsole et al. 2014) and is the causal agent of olive quick decline syndrome (OQDS) (Saponari et al. 2016). Since the initial outbreak, the disease has spread through the majority of the olive trees (*Olea europaea*) in Lecce province (23,000 ha) (EFSA PLH Panel (EFSA Panel on Plant Health) 2015). *X. fastidiosa* CoDiRO (referred to as *X. fastidiosa* hereafter) is spreading northward and is threatening olive production throughout Italy and beyond (Martelli 2015; Bosso et al. 2016a, b), and has attracted significant media attention (Abbott 2015; Nadeau 2015; Stokstad 2015). The *X. fastidiosa* bacterium is generally transmitted by various species of xylem-feeding bugs (Homoptera, Auchenorrhyncha), which are widespread (Elbeaino et al. 2014). Specifically, in olives in Apulia, Italy, *X. fastidiosa* is vectored by the froghopper *Philaenus spumarius* (Saponari et al. 2014b). Currently, there is no known cure for this deadly disease of olives and the only approaches to control are to destroy the host trees and create buffer zones around them or to manage the insect vector population by insecticides or removal of their weed habitats (European Union 2015).

The outbreak in southern Italy is characterised by extensive leaf scorch and dieback of olive trees, which has caused significant economic loss (Stokstad 2015). *X. fastidiosa* has a very broad range of known host plants in Europe, including many grown agriculturally, and hence the disease could have a large impact on food production (EFSA PLH Panel (EFSA Panel on Plant Health) 2015). Pierce’s disease in grapevines has been estimated to cost California $104.4 million per annum (Tumber et al. 2014), although it is difficult to infer the risks of *X. fastidiosa* in Europe because of the ecological and taxonomic complexity of this pathogen and the fact that the biota, as well as climatic conditions, in Europe are different from those in the Americas (EFSA PLH Panel (EFSA Panel on Plant Health) 2015).

Recently, specific and compulsory measures to control the *X. fastidiosa* epidemic have been designed and implemented (European Union 2015). The measures are based on an integrated pest management strategy that includes insecticide applications against the vector, agronomic measures to suppress nymphal stages of the vector on weeds and removal of infected and uninfected hosts. Demarcated areas and a buffer zone have been introduced across the peninsula to try and stop *X. fastidiosa* spreading further northward (European Union 2015). However, there are no data to suggest how well these countermeasures will perform. Thus the value of a predictive mechanistic model would be to provide some preliminary estimates of control effectiveness, which in turn may aid in determining whether a control policy needs to be improved or abandoned. In addition, the importance of olives for human livelihoods in the region means the strategy of removing diseased and healthy trees is extremely controversial (Abbott 2015), which has led to a disparity between legislation and implementation (Nadeau 2015).

While studies on this disease are ongoing, quantifiable data and measurements on its spread are scarce, and this is compounded by differences in the bacterial strain, host, vector and environment compared to *X. fastidiosa* infestations in other parts of the world (EFSA PLH Panel (EFSA Panel on Plant Health) 2015). Therefore, predicting the extent of spread and its impacts are extremely difficult, and hence assessing the efficacy of control measures are even more problematic. One approach to investigate the extent of potential spread of the disease is to use species distribution modelling (Hoddle 2004; Bosso et al. 2016a, b). This correlative approach uses statistical fitting to predict the potential distribution of species in geographic space on the basis of their known distribution in environmental space. As such, these static models fail to incorporate mechanisms of spread and thus cannot predict any spatial–temporal dynamics (e.g. where the disease may spread to at a future point in time) (Dormann et al. 2012). Conversely, detailed mechanistic models require known parameter values, but since the epidemiological data differs from previous *X. fastidiosa* outbreaks compared to the current outbreak, using past data in a detailed mechanistic spread models is likely to lead to misinformation. Simple statistical models of spread require spatio-temporal data for fitting (e.g. Gilbert et al. 2004) or directly-measured model parameters (e.g. Parnell et al. 2015), neither of which exist for the outbreak of *X. fastidiosa* in Apulia. Due to these data limitations we constructed a simple mechanistic model which is validated against the current spatial distribution of positive, laboratory-tested cases of the disease.

In this paper, we build upon a novel mechanistic model for the spread of *X. fastidiosa* in Apulia (Chapman et al. 2015) and show that it qualitatively and quantitatively fits the observed pattern of spread. Using the spread model we test the control strategies currently being employed, namely the eradication zone (EZ) and buffer zone (BZ). The efficacy of these control strategies are then discussed as well as their sensitivities to changes in control effort and surveillance efficiency, as well as the role of alternative hosts. Because this is an emerging disease, the parameter values used in the model are uncertain, and so the primary aim of this paper is to assess qualitatively the major processes likely to govern spread and effectiveness of control strategies. Quantitative predictions require better empirical data, and the model can also indicate which data are most critical.

## Methods

We model the spread of *X. fastidiosa* using a spatially explicit simulation model, building upon the spread model presented in Chapman et al. (2015) (see Case Study 5), which we briefly describe in the sections below. The model runs over the Apulian region at a 1 km^2^ gridded resolution and at a yearly temporal scale to correspond with the seasonality of the vector which only feeds on olive trees in the summer months when host grasses dry-out (EFSA PLH Panel (EFSA Panel on Plant Health) 2015). *X. fastidiosa* modelled spread has two distinct phases: local infection growth within a grid cell (i.e. progression of disease within the grid cell as a fraction of trees infected); and dispersal between grid cells. This approach is well suited to the underlying epidemiological mechanisms of vector spread and phenology (Chapman et al. 2015; EFSA PLH Panel (EFSA Panel on Plant Health) 2015). Chapman et al. (2015) showed that the model reproduces the qualitative patterns of *X. fastidiosa* spread in Apulia, Italy. In this paper, we use this framework to provide estimates of the accuracy of the underlying spread model by comparing risk analysis with spatial infection data. We then extend the model to include spatially explicit buffer zone control strategies across the Apulian peninsula which provide implementation guidelines for policy. Full Matlab code of this simulation model is available on GitHub (White et al. 2016).

### Local growth

To model the local infection growth in a grid cell we use a Gompertz equation to represent the fraction of infected host trees over time, denoted by *N*(*t*). Gottwald et al. (1993) studied the progression of citrus variegated chlorosis (CVC) in Brazil and found that a Gompertz model best fitted the progression data. The progression of *X. fastidiosa* infection in olive trees in Italy is thought to be much faster than that in citrus in Brazil, but we assume that the progression will have a similar sigmoidal shape. It should be noted that complex temperature/seasonal dynamics are likely to affect the disease incidence (Laranjeira et al. 2000), but this is currently unknown for OQDS. Hence, we assume a continuous time Gompertz equation is given by

1 $$N\left( t \right) = K{ \exp }\left[ { - B{ \exp }\left( { - At} \right)} \right].$$

The parameter *B* is related to the initial proportion of plants that are infected, *A* describes the rate of population growth (disease progression rate) and *K* is the carrying capacity (the maximum fraction of infected trees). To allow a local infection rate much faster than for CVC (Gottwald et al. 1993) (*A* = 0.489), we fix *A* = 3 and retain the initial infection from Gottwald et al. (1993) as B = 14.069, leading to 97% infection in a 1 km^2^ grid cell after 2 years, which is commensurate with initial surveys (Giuseppe Stancanelli and Maria Saponari pers. comm.).

Although this equation models the infection dynamics implicitly, it has been shown that this model gives a good fit to an explicit infection model and is therefore underpinned by a mechanistic individual-based model (Chapman et al. 2015). One may rescale Eq. 1 to a discrete annual time-scale to coincide with the vector phenology such that the fraction of infected hosts at year *t* and grid cell (*x*, *y*) is given by

2 $$N_{t + 1} \left( {x,y} \right) = K\left( {x,y} \right)\left( {\frac{{N_{t} \left( {x,y} \right)}}{{K\left( {x,y} \right)}}} \right)^{{e^{ - A} }} =\!{:}\,f\left( {N_{t} \left( {x,y} \right)} \right).$$

Although we are predominantly interested in infections of olive trees, there is evidence to suggest that a number of less abundant alternative host plants can become infected with *X. fastidiosa* (Saponari et al. 2013, 2014a; Martelli et al. 2015; Potere et al. 2015). While the distribution of olive trees is known, no such information is available for alternative hosts. Furthermore, the infection pathways and number and identity of all alternative hosts are not fully understood. To this end, we define the grid cell infection carrying capacity as *K*(*x*, *y*) = Φ(*x*, *y*) + *a*(1 − Φ(*x*, *y*)), where Φ(*x*, *y*) is the proportional cover per 1 km^2^ grid cell of olive trees and *a* ∈ [0, 1] is the carrying capacity in non-olive grove habitat, relative to that in olive groves. The proportional olive cover was estimated by counting the presence-absence of olives in the containing 0.01 km^2^ sub-cells, corrected for land surface area in the 1 km^2^ cell (0.01 km^2^ presence-absence data provided by InnovaPuglia SpA).

### Dispersal

**Fig. 1:**
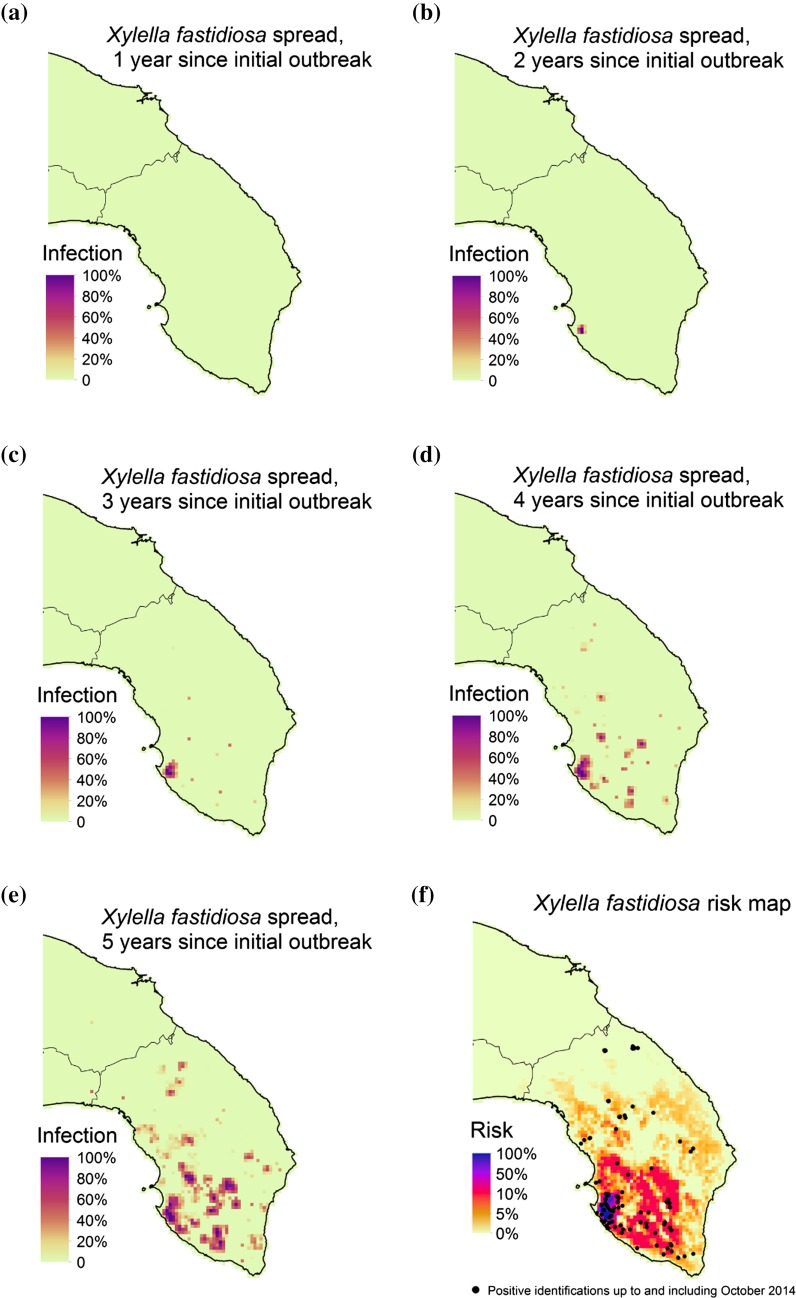
Typical model output from a single simulation of the model with stochastic long-distance dispersal. **a**–**e** The progression of the spread of *Xylella fastidiosa* throughout the region over 5 years, starting at a location close to Gallipoli, Apulia. *Darker red*/*purple colours* indicate high levels of infection within a patch. In **f** we plot the risk, defined as the average disease incidence from 10,000 stochastic model runs after 5 years, and the locations of positive tests for *X. fastidiosa* in olives. Positive test data was supplied by InnovaPuglia SpA, where the positive test was performed by using PCR assays and DAS-ELISA (Saponari et al. 2013)

While the mechanisms of *X. fastidiosa* dispersal are known [vectors disperse by flight locally or by wind, or are transported unintentionally by human vehicle movement (hitchhiking)], they have not been well quantified. The disease distribution (see Fig. 1f), suggests that from the suspected initial outbreak location near Gallipoli (the concentrated area of positive *X. fastidiosa* tests on the west of the peninsula) there has likely been a degree of local spread in conjunction with long-distance dispersal, resulting in a strong clustering of outbreak locations. This two-process dispersal is commonly reported and is known as stratified dispersal (Shigesada et al. 1995), which we model here.

We represented the short-distance dispersal of the insect vector with a deterministic 2D exponential dispersal kernel, with a mean dispersal distance of *β* km, for the local spread. In the absence of detailed dispersal data, the exponential is a good starting distribution to use in spread models (e.g. Neubert and Caswell 2000). The kernel is given by

$$\hat{k}\left( {x,y} \right) = \exp \left( { - \frac{{\left( {x^{2} + y^{2} } \right)^{1/2} }}{\beta }} \right).$$

Without greater knowledge on the local dispersal distance of *Philaenus spumarius*, the main vector of *X. fastidiosa* in Apulia (Saponari et al. 2014b), and how this translates into olive tree infection, we assume that the mean dispersal distance, *β*, is 100 m (Blackmer et al. 2004). It should be noted that a normalizing constant is not required for infection spread.

From the single snapshot of spread of *X. fastidiosa* it is impossible to characterise the nature of the random long-distance dispersal events (cf. Gilbert et al. (2004) for example, where human population density influences dispersal directionality of a human-transported species). For simplicity, we assume isotropic stochastic dispersal. We assume that dispersal into the sea is not possible since one of the main mechanisms of dispersal is hitchhiking on vehicles. We assign a weighted probability for each 1 km^2^ grid cell generating a random disperser, given by *ρN*_*t*_(*x*, *y*), where *ρ* ∈ ∪[0, 1]. Thus, grid cells that are heavily infected with *X. fastidiosa* will have a greater probability of generating a long-distance disperser. If the grid cell probability is greater than a threshold, *p* (Bernoulli trial), then a random number of dispersers, *M* ∈ {1, 2, …, *M*_max_}, disperse a random distance, given by a 2D discrete Gaussian distribution, *N*(0, *d*). Newly infected random grid cells have an initial infection level of *e* ^−*B*^, the initial infection level as described by the Gompertz equation.

There are three unknown parameters associated with long-distance dispersal (*p*, *M*_max_ and *d*), which cannot be parameterised from the spread data or existing literature. We explored a range of values for these parameters and selected reasonable values so that the modelled spread patterns resemble qualitatively that of the spread data (see “Results” section): *p* = 0.2, *M*_max_ = 5 and *d* = 20 km.

### Spatial model

The regional scale spread model can be written as

3 $$N_{t + 1} \left( {x,y} \right) = \mathop \sum \limits_{i = 1}^{n} \mathop \sum \limits_{j = 1}^{m} k\left( {x - i,y - j} \right)f\left( {N_{t} \left( {i,j} \right)} \right),$$

where *k* is the sum of the short-distance deterministic and long-distance stochastic kernels, and *f* is the growth function given by Eq. 2. To simulate Eq. 3, we may make use of convolution theory and the discrete fast Fourier transform (FFT) for a fast and efficient method (Allen et al. 2001).

### Control strategies

The European Commission audit (European Commission 2014) on the spread of *X. fastidiosa* in Italy proposed control measures to stop the northward spread of the disease which the European Union (EU) later approved (European Union 2015). The control efforts include roguing of infected plants, removing host plants, insecticide treatments to reduce vectors on both weed and olive plants, and removal of vector habitat. These approaches are aimed at preventing infection introduction and outbreak containment. Currently, there is no known eradication strategy, largely due to the broad host range of the pathogen and its vectors (EFSA PLH Panel (EFSA Panel on Plant Health) 2015). The affected demarked area (DA) is divided into four zones: infected zone (IZ), eradication zone (EZ), buffer zone (BZ) and surveillance zone (SZ), within which the control measures vary (see Appendix S2 for further details). Each zone spans the peninsula from the East to the West coasts. The EFSA opinion (EFSA PLH Panel (EFSA Panel on Plant Health) 2015) states that there can be no successful eradication of *X. fastidiosa* once it is established and therefore efforts should be concentrated on preventing infections in disease-free areas. Therefore, as a simplification and worst case scenario, we model the EZ and BZ control strategies, but assume that no control strategy is employed in the IZ (see Appendix S2). This approach allows us to concentrate on the efficacy of preventing northward spread rather than endemic disease reduction in concordance with the implemented control strategy (Martelli 2015).

We model the control in these zones by assigning a probability of infection detection, *p*_detect_ ∈ ∪[0, 1], each year to every grid cell within the zone that is infected, such that if the surveillance efficiency, *s* ∈ [0, 1], is greater than the detection probability (*s* > *p*_detect_) then those infected olive trees within the grid cell are removed and not replaced. If *s* = 1 then all infected olive trees are detected and removed without replanting; we refer to this as perfect control. Conversely, if *s* = 0 then there is no control strategy and *X. fastidiosa* may spread unimpeded. Furthermore, we assume that *p*_detect_ is independent of the level of infection since olive growers will inspect each tree and thus even small outbreaks may be detected. Once infection has been detected all of the infected hosts are removed/rogued (*N*_*t*_(*x*, *y*) = 0), and the carrying capacity is adjusted accordingly. Note that we may implicitly specify whether infected olive and/or non-olive hosts may be removed since the ratios of olives to non-olives are known via the carrying capacity equation.

## Results

### Spread model

We start our stochastic simulations to the south of Gallipoli, the suspected initial outbreak location (Martelli 2015). Typically, the initial spread is localised to the Gallipoli area (see Fig. 1a–e), but as time passes satellite infection sites occur, from which local spread occurs. This pattern repeats, creating hotspots of infection that are several km across, depending on the distribution of olive host plants (see Fig. S1).

Since the spread model is stochastic, we use risk maps (see Fig. 1f) to predict the probability that a location will become infected. Here we define risk as the average disease incidence from 10,000 stochastic model runs after 5 years from the initial outbreak near Gallipoli. We base the 5 year prediction horizon on the likely introduction time point to present (Martelli et al. 2015), although the exact arrival year of the disease is unknown (Donato Boscia pers. comm.). As expected, the areas closest to the disease epicentre are at highest risk with decreasing risk further away, but the model also predicts the risk is highly heterogeneous throughout the landscape due to the patchy distribution of host olive trees (see Fig. S1) and the distance from the epicentre. Comparing the risk map with positive tests for *X. fastidiosa* suggests that the model predicts the spread of the disease well qualitatively. To evaluate the model predictions quantitatively, we used the continuous Boyce index *B* as described by Hirzel et al. (2006) (see Appendix S2 for further details). This gave a value of *B* = 0.951 (B varies from −1 to 1; positive values indicate predictions correlate with the data; values close to zero indicate that the model is not different from a chance model; negative values indicate that the model does not correlate with the data), indicating a very strong correlation between the modelled risk and the observed disease outbreaks.

Increasing the rate of local infection (*A*) and the occurrence of random long-distance dispersal events (*p*, *M*_max_) leads to greater disease incidence and spread (see Chapman et al. 2015). While this remains true of the long-distance dispersal parameter (*d*), the effect on disease incidence is small. Thus, greater long-distance dispersal has little impact on the severity of the *X. fastidiosa* outbreaks, but will aid in its spread.

### Control strategies

**Fig. 2:**
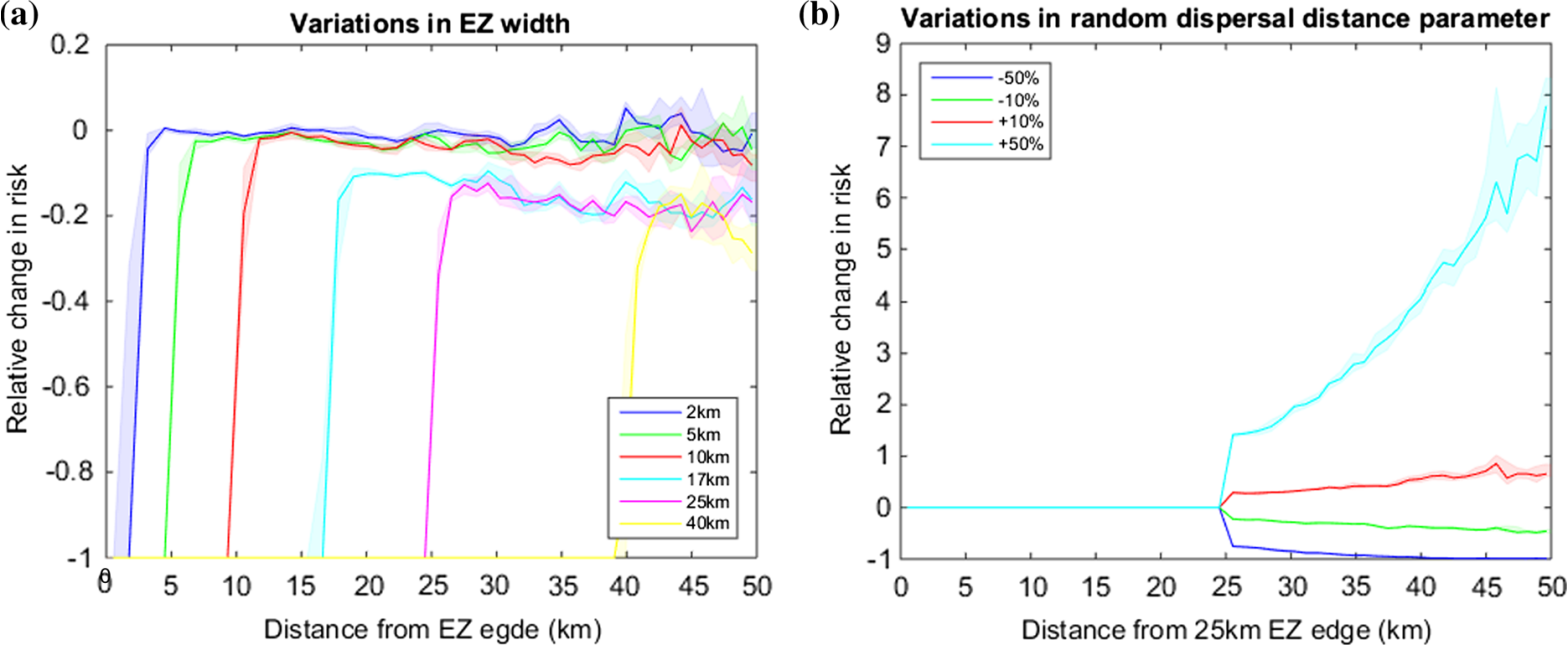
Modelling the risk associated with a perfect Control Zone (CZ). In **a** the relative risk is plotted for varying CZ widths. In **b** the relative risk is plotted for a 25 km CZ with varying long-distance dispersal distance parameters. Relative risk is calculated as ${\text{risk}}_{\text{rel}} = \frac{{{\text{risk}}_{\text{cont}}-{\text{risk}}_{\text{uncont}} }}{{{\text{risk}}_{\text{uncont}}}},$ where risk_cont_ and risk_uncont_ are the risks at each location for the controlled and uncontrolled scenarios respectively. In both plots each simulation is started from the known distribution of positive *X. fastidiosa* locations (see Figs. 1, 2) and repeated 10,000 times after which the risk is calculated. For each location beyond the starting line of the CZ the perpendicular distance is calculated from the line. All data is binned into 50 bins and smoothed with a moving mean to reduce stochasticity so that underlying trends are more apparent. The median line plot is plotted along with the shaded interquartile range. In **a** the relative change in risk within the CZ is −1

After showing that the spread model captures the qualitative dynamics of *X. fastidiosa* spread, we use the model for assessing the potential efficacy of control strategies. To begin our analysis of the EZ and BZ we assume that the surveillance intensity is the same in both zones, such that both have effectively the same control regime. We refer to this zone as the control zone (CZ). Furthermore, we assume that control within this zone is perfect. This is essentially the best case scenario where cost is no option. We vary the width of the CZ and plot the effects of the relative risk (the risk as compared to the risk where no control is applied) measured from the start of the CZ edge and extending northwards beyond the current disease distribution [see Fig. 2a and Appendix S3 Fig. S3 (a)]. Our analysis shows that for narrow CZ widths the risk is only reduced in the CZ, and beyond that the risk is largely unchanged from the no control scenario. This indicates that narrow CZ widths have little effect on protecting olive trees beyond the CZ and therefore are unlikely to stop the northward spread of *X. fastidiosa*. This is because the random long-distance dispersal simply jumps over the control zone. In contrast, for wider control zone widths, the reduction in risk is observed further away from the control zone and is therefore more likely to slow the spread of the disease.

Since the width of the CZ has a large effect on managing *X. fastidiosa* risk and that narrow zones fail to significantly reduce risk significantly, the modelled dispersal distance is likely to interact with this. In Fig. 2b we plot variations in the long-distance dispersal parameter (*d*) for a CZ of 25 km, as we previously established that *d* is one of the key mechanisms driving the rate of spread of *X. fastidiosa* (Chapman et al. 2015). The plot shows that the value of the long-distance dispersal parameter in relation to the CZ width is very important in determining whether the control strategy will reduce risk; small distances relative to the CZ width may reduce the risk to negligible levels, while large distances may increase the relative risk by orders of magnitude, especially for locations far beyond the CZ [also see Appendix S3 Fig. S3 (b)].

**Fig. 3:**
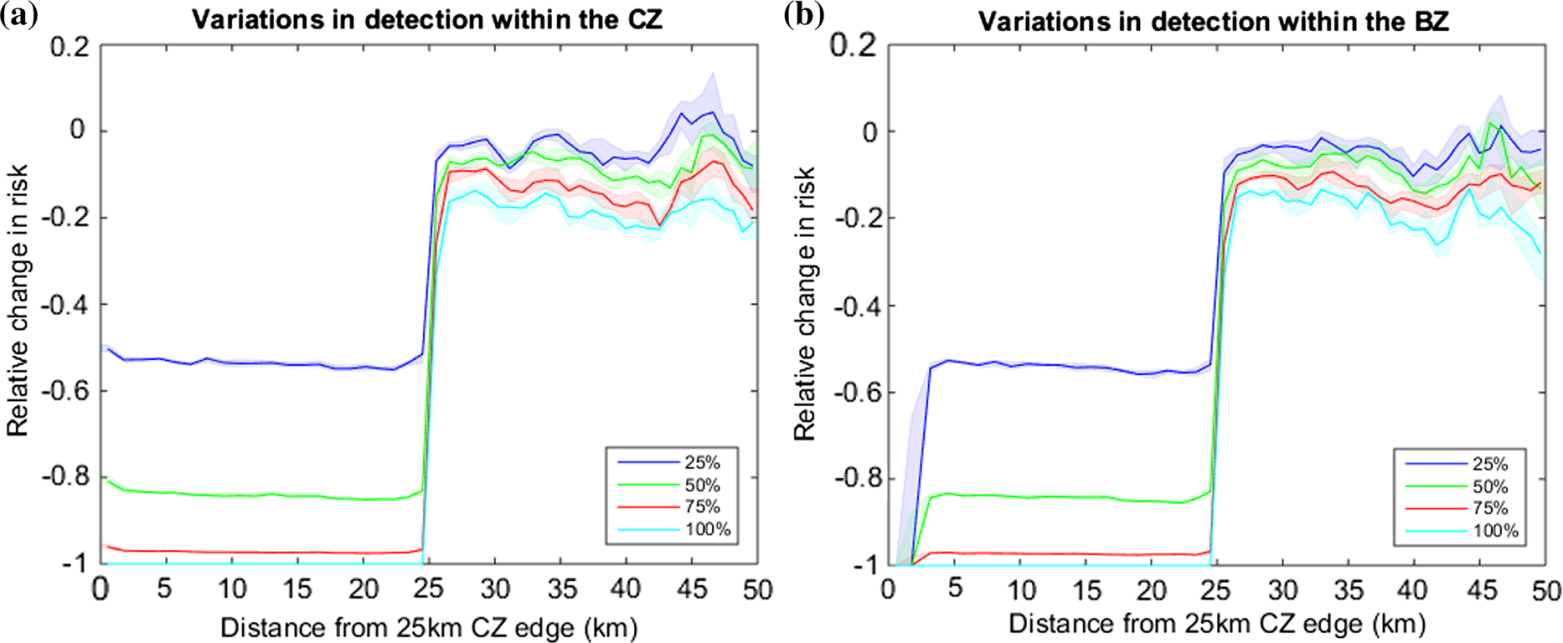
Determining the effects of surveillance effort on risk. In **a** we plot the relative risk as we vary the surveillance effort, *s*, within a 25 km CZ. In **b** we plot the relative risk as we vary the surveillance effort within a 23 km BZ which precedes a 2 km perfect EZ. The relative changes in risk are calculated by comparing the controlled and uncontrolled scenarios. All other parameters and interpretations are as in Fig. 2

The effort required for detecting *X. fastidiosa* over a large region such as the CZ is substantial. Furthermore, given the current state of knowledge on the disease in olive hosts, there may be a significant lag between initial infection and disease symptoms being expressed (EFSA PLH Panel (EFSA Panel on Plant Health) 2015), which might allow the infection to go undetected and cause further spread. Here we use our model to predict the effects of surveillance effort and detection within the zones. To this end, we vary the surveillance efficiency parameter, *s*, as a proxy for intensity of searching within the CZ. Our results (Fig. 3 and Appendix S3 Fig. S4) suggest that the relative risk is equally reduced within the CZ for all surveillance efficiencies (almost horizontal lines within the CZ), but the level of control is reduced as the efficiency is reduced. This effect is commuted beyond the CZ, although the relative changes in risk are narrowed. The qualitative behaviour is replicated even when there are different detection efficiencies in the EZ and BZ (see Fig. 3b). Comparing these figures suggests that having increased surveillance in a small EZ compared to the surveillance in the BZ has little effect on preventing the northward spread of *X. fastidiosa*.

### Alternative hosts

**Fig. 4:**
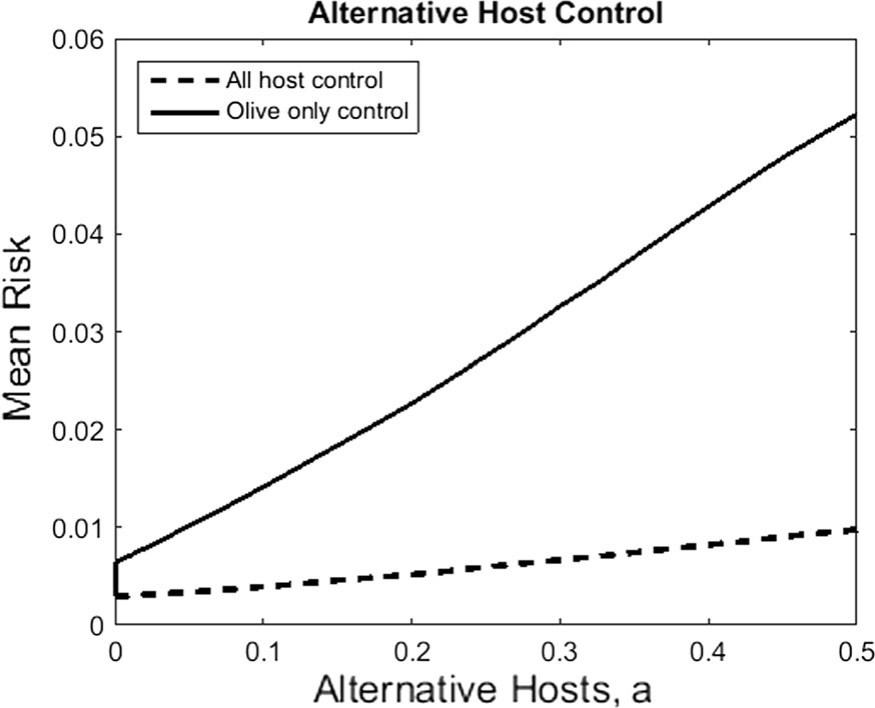
The effects of alternative hosts on the efficacy of control strategies. In this figure we plot the mean risk (the mean of the risk as defined in Fig. 2) for varying values of alternative hosts. We consider two 25 km CZ control strategies: one where only olive hosts are controlled; and one where olive and alternative hosts are controlled. Within the CZ it is assumed that the control strategy is perfect in that infected hosts are immediately discovered and removed. All other parameters are as in Fig. 2

Previous sensitivity analysis of the model demonstrated that the rate of spread of *X. fastidiosa* may be highly sensitive to the abundance of alternative hosts plants (Chapman et al. 2015). To ascertain how alternative host plants may potentially affect control effectiveness we consider the mean risk across the uninfected area of Apulia as the abundance of alternative hosts varies (Fig. 4). Furthermore, we compare two control strategies: one where control is applied to the olive hosts only; and one where all hosts (olive and non-olive) are controlled. In both cases, as the abundance of alternative hosts increases then so does the risk. However, controlling all hosts markedly improves reduction in risk (up to eightfold), which becomes more pronounced as the abundance of alternative hosts increases. This highlights the importance of alternative host identification, their role in *X. fastidiosa* spread and control.

### Optimization

Our results suggest that increasing either the width of the CZ or the surveillance within it will reduce the risk of the northward spread of *X. fastidiosa* (Figs. 2, 3). Given that resources to tackle the spread of the disease are limited, it is natural to ask whether it is beneficial to invest in greater CZ widths or surveillance, and how this depends on the total amount of resource.

**Fig. 5:**
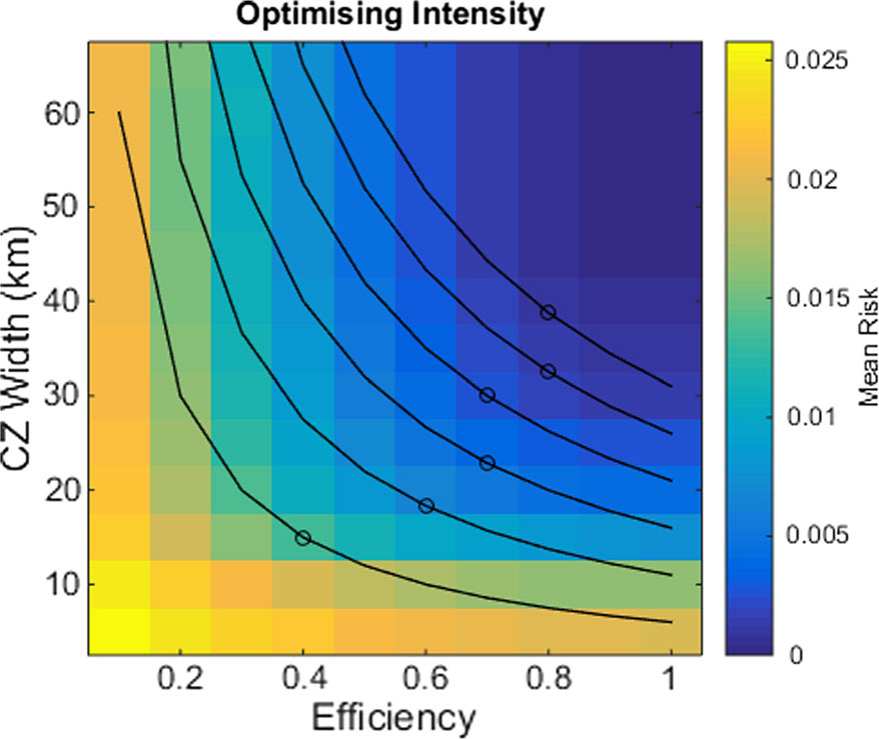
Variations in CZ width and surveillance effort on the mean risk across the spatial domain for a 25 km CZ. We covary the CZ width and surveillance efficiency, s, for a 25 km CZ and calculate the mean risk across the spatial domain beyond the CZ edge, denoted by the *colours* (*blue* denotes low risk; *yellow* denotes high risk). *Black lines* indicate contours of equal intensity (higher intensities appear in the *top right corner* of the plot) and *black circles* indicate their optimal value where the mean risk is minimised for the given intensity. The minima are calculated by varying the width and surveillance efficiency parameters along the contours and calculating the corresponding mean risk

To this end, we covary the CZ width with the surveillance efficiency and calculate the mean risk across the domain beyond the CZ edge (Fig. 5). We define the intensity as the product of the CZ width and surveillance efficiency, and thus serves as a proxy to the control strategy budget; higher intensity permits greater combinations of widths and efficiencies, as depicted by the black contours. Thus we may vary along the contour combinations of widths and efficiencies to find where the risk is minimised, and thus providing an optimal control combination for a given intensity budget.

Modelled simulations predict that the greatest reduction in risk is achieved with highly efficient and wide control zones (Fig. 5). However, under budget constraints, the risk can be minimised by non-extreme CZ widths or detection efficiencies. Furthermore, changing the budget changes the optimal control values. Our model predicts that optimal CZ strategies should concentrate on increasing searching efficiency for low budgets, but this should change to increasing CZ widths for larger budgets.

## Discussion

The rapid invasion of *X. fastidiosa* causing OQDS in Italy is causing substantial damage to olive production and the local economy, and is extremely worrying for neighbouring olive-producing regions in Italy and in other Mediterranean regions (Bosso et al. 2016a, b). Predicting its spread is important, since this will help guide control strategies and assess risk, and existing statistical distribution models (Bosso et al. 2016a, b) only predict the potential spatial extent of the disease, not the rate and patterns of spread or impacts of spatial control approaches. Here, we have developed a simple model based on previous work (Chapman et al. 2015) which we have compared to the known spatial and temporal dynamics of *X. fastidiosa* and shown that the model qualitatively reproduces the pattern and speed of spread of *X. fastidiosa* in the Apulian region. It should be noted that the data presented here provides a single snapshot of the spread of infection to which we have used a single measure (Boyce index) to validate the model. Furthermore, there may have been a period of time where *X. fastidiosa* spread which went undetected. Stronger validation would be available with sequential of infection data from the point of initial outbreak, but this is not currently available. Moreover, by the time such data are available, *X. fastidiosa* may have spread beyond the current infected area if left unchecked, causing catastrophic damage in the process, and may not be stoppable, as found with other plant pathogens such as sudden oak death (Cunniffe et al. 2016). Hence, we have provided the first attempt to model the spread of the disease in the early stages of invasion in Apulia with the aim of understanding generic mechanisms of spread and to elucidate upon control strategies.

The control strategies available to prevent the northward spread of *X. fastidiosa* are mostly based on infected and prophylactic host destruction through buffer zones and vector control (EFSA PLH Panel (EFSA Panel on Plant Health) 2015). However, the effectiveness of such control strategies are likely to depend on the underlying ecology and extent of the control method. The European Union decision on preventing the spread of *X. fastidiosa* states that the width of the buffer zone should be calculated in view of the risk of spread to other areas (European Union 2015). We have shown the width of the zone is crucially important in reducing the risk of northward spread of the disease, with buffer zones that are not sufficiently wide in relation to dispersal distances (specifically the rare, stochastic long-distance dispersal distances) having a relatively negligible effect on reducing the risk of spread.

In general, modelling approaches are useful tools for guiding risk assessments and for mitigating against invasive plant diseases (Chapman et al. 2015; Bosso et al. 2016a, b). For example, new techniques have been developed to guide surveillance strategies for emerging plant diseases (Parnell et al. 2014) or in predicting their spread (see Chapman et al. (2015) for a review). These techniques often rely on known parameter values, such as growth rates (Parnell et al. 2015), to make future predictions. However, there are significant issues with using these predictive models for emerging diseases as opposed to re-emerging or endemic diseases, namely the lack of empirical data to inform parameter values. In the case of *X. fastidiosa*, the outbreak in Apulia is a different strain to previous outbreaks, infects different hosts and experiences different environments (EFSA PLH Panel (EFSA Panel on Plant Health) 2015). Hence, parameters derived from past outbreaks may not be relevant or may lead to erroneous predictions. To deal with this uncertainty, our approach is to use a simple model and qualitatively validate the predictions against current spread data, but it is clear that more accurate predictions would be possible if relevant parameter values where available. Therefore, we advocate that field estimates of key parameters, such as infection growth rates, local and non-local dispersal parameters, asymptomatic infection lag and host range, be estimated post-haste. This will not only allow better predictive models, but also inform current and future control strategies, including surveillance (Parnell et al. 2015). However, our model is of immediate use in helping understand the spread and inform the control of *X. fastidiosa*. As global change accelerates, there is a need to undertake actions rapidly to counter the emerging negative impacts, even while data to inform these decisions may be limited (Shea et al. 2014). Approaches to addressing this conflict involve iterative decision making and adaptive management, whereby actions are modified as new information becomes available (Polasky et al. 2011), for example disease management is updated as research provides more certain model parameters.

While the mechanisms built into the model represent the key behaviours, more complex mechanisms may also occur that affect the growth and spread of OQDS. For example, seasonal or spatial factors may affect disease incidence, as was shown for CVC (Laranjeira et al. 2000). Temperature also regulates the dynamics of *X. fastidiosa* bacteria in grapevines, which can limit its potential distribution (Hoddle 2004). However, it is unknown how the bacterium is affected by temperature in olive hosts. Despite this, attempts have been made to map the potential distribution of OQDS in the Mediterranean basin, estimating high suitability for the disease throughout the modelled region (Bosso et al. 2016a, b). Also, as disease causes tree die-back, the levels of infection may also change. However, it is likely that this will only affect the infection levels in the infected zone and will not alter the rate of spread, since spread rates are usually determined by the infection levels at the front of the expanding infection (Neubert and Caswell 2000), but this will be dependent on the interaction between the rate of die-back and the dispersal mechanisms. Including these complexities is not justifiable without additional supporting data.

Given the paucity of data, our model has provided useful insight into the spread of *X. fastidiosa* and potential control strategies. Our analysis predicts that the long-distance dispersal events are an extremely significant factor in the rapid spread of *X. fastidiosa* and therefore targeting control measures at this mechanism would be highly advantageous. Reducing vector numbers through insecticide application or weed control will certainly aid in reducing the probability of long-term dispersal events, but preventing vectors hitchhiking on vehicles will be more challenging. However, raising public awareness of the disease could encourage vehicle checks, akin to the “Check, Clean, Dry” campaign for preventing the spread of aquatic invasive species in the UK (Non-native Species Secretariat 2016), may aid in reducing vector dispersal. Even if these measures are implemented, our model predicts that creating wide buffer zones may not completely eliminate the risk of spread of disease beyond the control zone. Nevertheless, the effectiveness of spread reduction is highly dependent on the underlying epidemiology and ecology of *X. fastidiosa* spread in Apulia, which is not well quantified (EFSA PLH Panel (EFSA Panel on Plant Health) 2015). In particular, it is critical to quantify stochastic long-distance dispersal events, which are only likely to be achieved by detailed landscape scale surveillance. Furthermore, since vector hitchhiking may be a main mechanism of the long-distance stochastic dispersal, then it stands to reason that is not isotropic due to the distribution of the road networks, traffic flows and human population densities in the region. These factors have been shown to be important in the spread of other invasive species (Gilbert et al. 2004).

While olive trees have been most significantly impacted by *X. fastidiosa* in Italy, other host plants may also aid the spread of the bacterium, including oleander (*Nerium oleander*), almond (*Prunus dulcis*), myrtle-leaf milkwort (*Polygala myrtifolia*) and coastal rosemary (*Westringia fruticosa*) (Saponari et al. 2013, 2014a). Since potential vectors of *X. fastidiosa* are numerous and widespread (Elbeaino et al. 2014), it is likely that these alternative host plants may aid in the spread of the disease, as our results suggest. Furthermore, our model suggests that if infected alternative hosts are not controlled then the risk to uninfected regions may increase up to eightfold, depending on the abundance of alternative host plants. Hence, the identification of alternative host plants, their ability to spread the bacterium and their distribution, is paramount, especially if these hosts are asymptomatic and go undetected by visual surveys. This should be achieved by further field trials and experiments.

Destroying olive trees to control the spread of *X. fastidiosa* in Apulia is very costly to the grower (Abbott 2015; Stokstad 2015). Therefore cost-efficient control strategies are required. We have shown that optimal strategies exist that trade-off the balance of surveillance and extent of the control zones to minimise the risk of infection in uninfected regions of Apulia and beyond (Fig. 5), and that these strategies vary according to the budget available; shifting the focus of control efforts from searching to control extent as the budget increases. The logistics of shifting this effort may of course be problematic, given that the only method of control currently available are tree removal and vector control, although new methods, such as the use of endophytic bacteria (Lacava et al. 2004; POnTE 2015), may change this scenario.

The control strategies modelled here only occur with demarcated zones, reflecting current approaches, but in practice surveillance and control may occur beyond such a zone, especially if there is long-distance dispersal. Including these surveillance strategies into our model may alter the optimal strategy and our preliminary conclusions. Furthermore, models that aim to improve surveillance strategies rely upon accurate spread models to predict the locations of outbreaks (e.g. Parnell et al. 2015). Using overly simplistic spread models, or models that do not capture the mechanisms of spread (e.g. assuming diffusive dispersal with no long-distance jumps), to inform surveillance models may result in erroneous predictions that are counterproductive. However, developing complex models may require significant development and validation time, thus negating their usefulness in combatting emerging infectious diseases. Hence, the method we have undertaken, in developing a simple mechanistic model that can be qualitatively validated against preliminary spatial data, may prove useful in breaking the circular problem, despite the paucity of empirically determined parameters.

Our novel modelling strategy has highlighted the importance of several key of parameters and processes of the *X. fastidiosa* outbreak that are either unknown or not quantified. Much of the current research on the *X. fastidiosa* outbreak in Apulia is focusing on the disease transmission, genetics, monitoring, surveillance, and control methods (POnTE 2015). However, our model and sensitivity analyses highlight that research should also be focused on quantifying local and long-distance dispersal. This will allow better predictions of future spread and also guidance on the extent and effectiveness of control methods.

## Electronic supplementary material

Below is the link to the electronic supplementary material.

Supplementary material 1 (DOCX 971 kb)

## References

[CR1] Abbott A (2015). Scientists blamed for olive-tree ruin. Nature.

[CR2] Allen JC, Brewster CC, Slone DH (2001). Spatially explicit ecological models: a spatial convolution approach. Chaos Solitons Fractals.

[CR3] Baker CM, Bode M (2016). Placing invasive species management in a spatiotemporal context. Ecol Appl.

[CR4] Blackmer JL, Hagler JR, Simmons GS (2004). Comparative dispersal of *Homalodisca coagulata* and *Homalodisca liturata* (Homoptera: Cicadellidae). Environ Entomol.

[CR5] Bosso L, Di Febbraro M, Cristinzio G (2016). Shedding light on the effects of climate change on the potential distribution of *Xylella fastidiosa* in the Mediterranean basin. Biol Invasions.

[CR6] Bosso L, Russo D, Di Febbraro M (2016). Potential distribution of *Xylella fastidiosa* in Italy: a maximum entropy model. Phytopathol Mediterr.

[CR7] Chapman DS, White SM, Hooftman DAP et al (2015) Inventory and review of quantitative models for spread of plant pests for use in pest risk assessment for the EU territory. EFSA supporting publication 2015:EN-795, p 190

[CR8] Cunniffe NJ, Cobb RC, Meentemeyer RK et al (2016) Modeling when, where, and how to manage a forest epidemic, motivated by sudden oak death in California. Proc Natl Acad Sci USA 113:5640–5645 10.1073/pnas.1602153113 PMC4878485 27140631

[CR9] Dehnen-Schmutz K, Holdenrieder O, Jeger MJ (2010). Structural change in the international horticultural industry: some implications for plant health. Sci Hortic.

[CR10] Dormann CF, Schymanski SJ, Cabral J (2012). Correlation and process in species distribution models: bridging a dichotomy. J Biogeogr.

[CR11] Efsa, PLH Panel (EFSA Panel on Plant Health) (2015). Scientific opinion on the risks to plant health posed by *Xylella fastidiosa* in the EU territory, with the identification and evaluation of risk reduction options. EFSA J.

[CR12] Elbeaino T, Yaseen T, Valentini F (2014). Identification of three potential insect vectors of *Xylella fastidiosa* in southern Italy. Phytopathol Mediterr.

[CR13] European Commission (2014) Final report of an audit carried out in Italy from 10 to 14 February 2014 in order to evaluate the situation and official controls for *Xylella fastidiosa*. In: http://ec.europa.eu/food/fvo/audit_reports/details.cfm?rep_id=3285#

[CR14] European Union (2015) Commission implementing decision (EU) 2015/789 of 18 May 2015 as regards measures to prevent the introduction into and the spread within the union of *Xylella fastidiosa* (Wells et al.) (notified under document C(2015) 3415) In: http://eur-lex.europa.eu/legal-content/EN/TXT/?uri=CELEX:32015D0789

[CR15] Gautam HR, Bhardwaj ML, Kumar R (2013). Climate change and its impact on plant diseases. Curr Sci.

[CR16] Gilbert M, Gregoire JC, Freise JF (2004). Long-distance dispersal and human population density allow the prediction of invasive patterns in the horse chestnut leafminer *Cameraria ohridella*. J Anim Ecol.

[CR17] Gottwald T, Gidtti F, Santos J et al (1993) Preliminary spatial and temporal analysis of citrus variegated chlorosis in Brazil. In: Proceedings, 12th IOCV Conference, Riverside

[CR18] Hirzel AH, Le Lay G, Helfer V (2006). Evaluating the ability of habitat suitability models to predict species presences. Ecol Model.

[CR19] Hoddle MS (2004). The potential adventive geographic range of glassy-winged sharpshooter, *Homalodisca coagulata* and the grape pathogen *Xylella fastidiosa*: implications for California and other grape growing regions of the world. Crop Protect.

[CR20] Hulme PE (2009). Trade, transport and trouble: managing invasive species pathways in an era of globalization. J Appl Ecol.

[CR21] Lacava PT, Araujo WL, Marcon J (2004). Interaction between endophytic bacteria from citrus plants and the phytopathogenic bacteria *Xylella fastidiosa*, causal agent of citrus-variegated chlorosis. Lett Appl Microbiol.

[CR22] Laranjeira FF, Gottwald TR, Amorim L et al (2000) Spatio-temporal dynamics of citrus variegated chlorosis: a preliminary analysis. In: Proceedings 14th IOCV conference, Campinas, São Paulo, pp 223–231

[CR23] Loconsole G, Potere O, Boscia D (2014). Detection of *Xylella fastidiosa* in olive trees by molecular and serological methods. J Plant Pathol.

[CR24] Martelli GP (2015). The current status of the quick decline syndrome of olive in southern Italy. Phytoparasitica.

[CR25] Martelli GP, Boscia D, Porcelli F (2015). The olive quick decline syndrome in south-east Italy: a threatening phytosanitary emergency. Eur J Plant Pathol.

[CR26] Nadeau BL (2015). The battle of olives. Sci Am.

[CR27] Neubert MG, Caswell H (2000). Demography and dispersal: calculation and sensitivity analysis of invasion speed for structured populations. Ecology.

[CR28] Non-native Species Secretariat (2016) Check, clean, dry. In: http://www.nonnativespecies.org/checkcleandry/

[CR29] Parnell S, Gottwald TR, Riley T (2014). A generic risk-based surveying method for invading plant pathogens. Ecol Appl.

[CR30] Parnell S, Gottwald TR, Cunniffe NJ (2015). Early detection surveillance for an emerging plant pathogen: a rule of thumb to predict prevalence at first discovery. Proc R Soc Lond Ser B Biol Sci.

[CR31] Pimentel D, Zuniga R, Morrison D (2005). Update on the environmental and economic costs associated with alien-invasive species in the United States. Ecol Econ.

[CR32] Polasky S, Carpenter SR, Folke C (2011). Decision-making under great uncertainty: environmental management in an era of global change. Trends Ecol Evol.

[CR33] POnTE (2015) Pest organisms threatening Europe. In: http://www.ipsp.cnr.it/projects/ponte

[CR34] Potere O, Susca L, Loconsole G (2015). Survey for the presence of *Xylella fastidiosa* subsp. pauca strain CoDiRO in some forestry and ornamental species in the Salento peninsula. J Plant Pathol.

[CR35] Purcell AH, Hopkins DL (1996). Fastidious xylem-limited bacterial plant pathogens. Annu Rev Phytopathol.

[CR36] Richter R, Berger UE, Dullinger S (2013). Spread of invasive ragweed: climate change, management and how to reduce allergy costs. J Appl Ecol.

[CR37] Saponari M, Boscia D, Nigro F (2013). Identification of DNA sequences related to *Xylella fastidiosa* in oleander, almond and olive trees exhibiting leaf scorch symptoms in Apulia (Southern Italy). J Plant Pathol.

[CR38] Saponari M, Boscia D, Loconsole G (2014). New hosts of *Xylella fastidiosa* strain codiro in Apulia. J Plant Pathol.

[CR39] Saponari M, Loconsole G, Cornara D (2014). Infectivity and transmission of *Xylella fastidiosa* by *Philaenus spumarius* (Hemiptera: Aphrophoridae) in Apulia, Italy. J Econ Entomol.

[CR40] Saponari M, Boscia D, Altamura G et al (2016) Pilot project on *Xylella fastidiosa* to reduce risk assessment uncertainties. EFSA supporting publication 2016:EN-1013, p 60

[CR41] Shea K, Tildesley MJ, Runge MC (2014). Adaptive management and the value of information: learning via intervention in epidemiology. PLoS Biol.

[CR42] Shigesada N, Kawasaki K, Takeda Y (1995). Modeling stratified diffusion in biological invasions. Am Nat.

[CR43] Simberloff D, Martin JL, Genovesi P (2013). Impacts of biological invasions: what’s what and the way forward. Trends Ecol Evol.

[CR44] Stokstad E (2015). Food security. Italy’s olives under siege. Science.

[CR45] Tumber KP, Alston JM, Fuller KB (2014). Pierce’s disease costs California $104 million per year. Calif Agric.

[CR46] White SM, Bullock JM, Hooftman DAP et al (2016) *Xylella fastidiosa* spread model. doi:10.5281/zenodo.192974

