# Supplementary material for: Modelling the spread and control of Xylella fastidiosa in the early stages of invasion in Apulia, Italy
Source: Biol Invasions. 2017 Feb 21;19(6):1825–37. doi: 10.1007/s10530-017-1393-5 (PMC6979717; doi:10.1007/s10530-017-1393-5)
Supplement: Supplementary file 1 — Supplementary material 1 (DOCX 971 kb) [file 10530_2017_1393_MOESM1_ESM.docx]

Modelling the spread and control of Xylella fastidiosa in the early stages of invasion in Apulia, Italy: Supplementary Material

Steven M. White^1,2*^, James M. Bullock^1^, Danny A. P. Hooftman^1,3^ & Daniel S. Chapman^4^

^1^Centre for Ecology & Hydrology, Benson Lane, Crowmarsh Gifford, Wallingford, Oxfordshire, OX10 8BB, UK.

^2^Mathematical Institute, University of Oxford, Andrew Wiles Building, Radcliffe Observatory Quarter, Woodstock Road, Oxford, Oxfordshire, OX2 6GG, UK.

^3^Lactuca: Environmental Data Analyses and Modelling, Diemen, 1112NC, The Netherlands.

^4^Centre for Ecology & Hydrology, Bush Estate, Penicuik, Midlothian, EH26 0QB, UK.

^*^Corresponding author: email smwhit@ceh.ac.uk; telephone +44 1491 692699; fax +44 1491 692424

**Appendices**

*S1. Distribution of olives in Apulia*

The proportional olive cover was estimated by counting the presence-absence of olives in the containing 0.01 km^2^ sub-cells, corrected for land surface area in the 1 km^2^ cell (0.01 km^2^ presence-absence data provided by InnovaPuglia SpA). The result of this calculation is presented in Fig. S1 below.


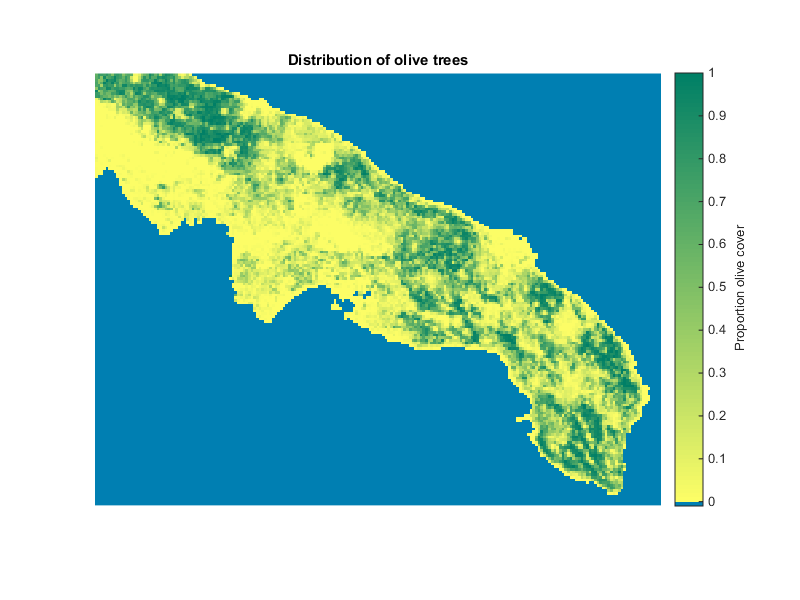


Figure 1: Distribution of olive cover in Apulia. Proportion cover was calculated at a 1 km^2^ resolution from 0.01 km^2^ presence absence data. Yellow colours indicate low proportions of olive cover and green colours indicate high proportions.

*S2. Quantitative validation of the spread model*

To evaluate the model predictions, we used the continuous Boyce index *B* as described by Hirzel et al. (2006). This index was developed to evaluate the output of presence-only species distribution models, in which a habitat suitability score is contrasted with observed occurrences of a species. Here, we applied it to assess the correspondence between predicted average disease risk and the observed outbreaks. *B* varies between -1 and 1, with higher values indicating a strong correspondence between the modelled favourability and the observed presences.

Our evaluation was restricted to the 2861 grid cells within 20 km of the observed detections and with olive cover > 0%. This avoided over-estimation of model performance by inclusion of locations very distant from the epidemic or unsuitable for *X. fastidiosa* colonisation.

Because our modelled values of average risk were highly skewed towards zero, we converted the risk values into more uniformly-distributed favourability scores *F*, using a transformation suggested by Real et al. (2006):

$F=\frac{\frac{R}{1-R}}{\frac{n_{1}}{n_{0}}+\frac{R}{1-R}}$.

Here, *R* is the modelled risk, $n_{1}$ is the number of grid cells with *X. fastidiosa* detections in the evaluation region and $n_{0}$ is the number with no *X. fastidiosa* detection. The transformation preserves the rank ordering of risk.

The Boyce index *B* was then calculated for the favourability scores, using 1000 evenly spaced focal windows with a width of 0.1 favourability units. This gave *B* = 0.951, indicating a very strong correlation between the modelled risk and the observed disease outbreaks.

*S3. Control Strategies*

The EC audit (European Commission 2014) on the spread of *X. fastidiosa* in Italy proposed control measures to stop the northward spread of the disease which the EU later approved (European Union 2015). The affected Demarcated Area (DA) is divided into four areas: Infected Zone (IZ), Eradication Zone (EZ), Buffer Zone (BZ) and Surveillance Zone (SZ). Each zone spans the peninsula from the East to the West coasts (see Fig. 2 of European Commission (2014)). In the BZ all host plants (i.e. all species known to act as a host for *X. fastidiosa*) along the roads, ditches, canals etc must be removed, shredded and disposed of, to prevent them from acting as conduits of further transmission. In addition, phytosanitary treatments (e.g. insecticides) target both the vectors on weeds and adult vectors on olive and fruit trees. This strategy is also employed in the EZ, but in addition, all infected plants (symptomatic and laboratory confirmed) are eliminated, together with plants identified as probably infected. In contrast, in the SZ, treatments target vectors on weeds as well as the adult stages of the vector on the olive and other fruit trees. A strict host plant transportation ban is imposed in the IZ along with the destruction of infected trees. Within each of the zones surveillance for new infections is conducted with the most intense surveillance in the EZ in an attempt to halt the northward spread of *X. fastidiosa*.

The EFSA opinion (EFSA PLH Panel (EFSA Panel on Plant Health) 2015) states that there can be no successful eradication of *X. fastidiosa* once it is established and therefore efforts should be concentrated on preventing infections in disease-free areas. Therefore, as a simplification and worst case scenario, we model the EZ and BZ control strategies, but assume that no control strategy is employed in the IZ. This approach allows us to concentrate on the efficacy of preventing northward spread rather than endemic disease reduction.

In Fig. S2 we plot the olive density map with the Eradication Zone (EZ - black) and Buffer Zone (BZ - grey) superimposed on top. The definitions of the control efforts in each of the zones is described in the main text. To the south of the EZ is the Infected Zone (IZ) and to the north of the BZ lies the Surveillance Zone (SZ).

The precise location of the modelled EZ and BZ may differ to that which is currently being employed. However, our focus is on the efficacy of the control strategy and how it relates to the dispersal scales, the widths of the zones and the surveillance intensity within each zone. In doing this, we aim to provide a guide on how effective the control strategy is likely to be, given the assumptions of the model.


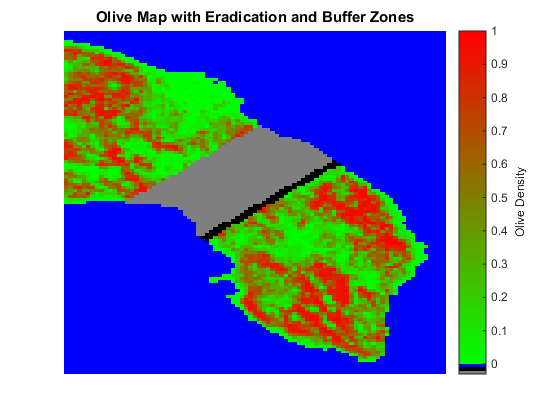


Figure S2: Map of olive trees and an example of the Eradication (EZ) and Buffer (BZ) Zones. The density of olive trees are calculated as described in the main text, with low density plotted in green and high density in red. Blue denotes the Mediterranean Sea. The EZ and BZ control strategies are shown in black and grey respectively. Here the EZ has a width of 2km and the BZ a width of 20km.

In Fig. S2 we plot the absolute changes in risk, as opposed to the relative changes in risk as presented in Fig. 3 of the main text. In (a) we plot a combined EZ and BZ (CZ) and assume that they both have the same perfect protection. The black, 0km, zone is the no control plot of risk. It shows that, in general, risk is reduced as one moves northward from the IZ. However, there is a great deal of heterogeneity in risk which are caused by the heterogeneous distribution of olive trees. In (b) we plot the effects on the risk for varying random dispersal distance parameters with a 25km CZ. The plot shows that increasing the distance parameter significantly increases the risk, even for locations far beyond the CZ.

| (a)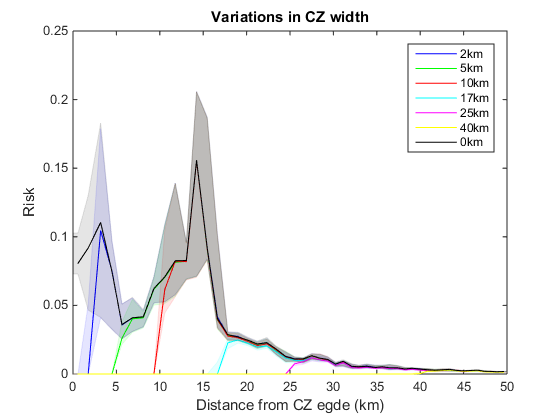 | (b)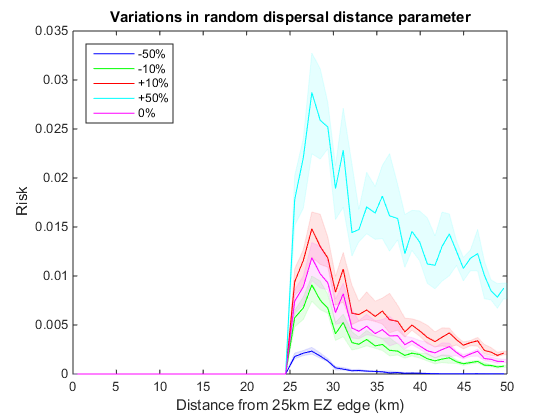 |
| --- | --- |

Figure S3: Modelling the risk associated with a perfect Control Zone (CZ). In (a) the risk is plotted for varying CZ widths. In (b) the risk is plotted for a 25km CZ with varying random dispersal distance parameters. In both plots each simulation is started from the known distribution of positive X. fastidiosa locations (see Figs 1 & 2 in the main text) and repeated 10000 times after which the risk is calculated. For each location beyond the starting line of the CZ the perpendicular distance is calculated from the line. All data is binned into 50 bins and smoothed with a moving mean to reduce stochasticity for that underlying trends are more apparent. The median line plot is plotted along with the shaded interquartile range. Also see Fig. 3 in the main text where we plot the relative changes in risk.

In Fig. S4 we plot variations in the surveillance efficiency parameter, $s$, as a proxy for intensity of searching within the CZ. In (a) we plot changes in risk for different surveillance efforts, $s$, in a 25km CZ. The plot shows that the risk is reduced within the CZ, but is only marginally reduced beyond the CZ (see Fig. 4 in the main text showing the relative changes in risk). In (b) we plot the results of varying s in a 23km BZ that is preceded by a 2km perfect EZ. Comparing these figures suggests that having increased surveillance in a small EZ compared to the surveillance in the BZ has little effect on preventing the northward spread of *X. fastidiosa*.

| (a) 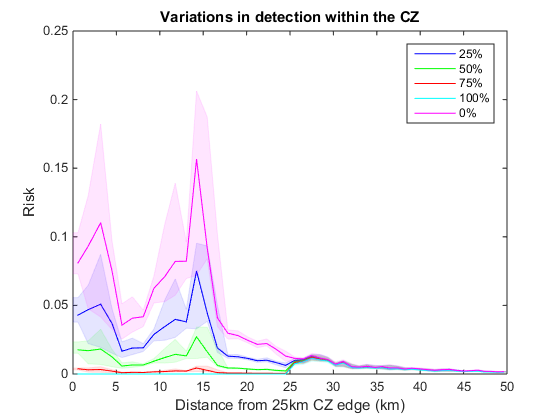 | (b) 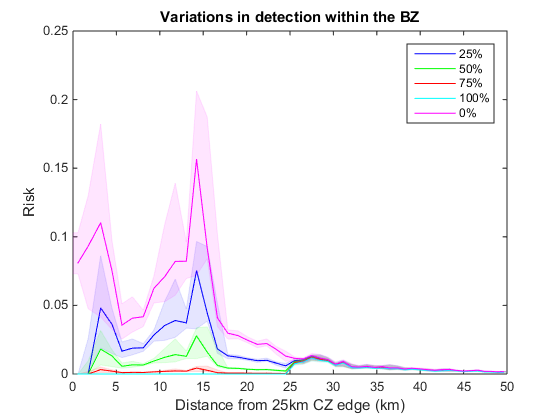 |
| --- | --- |

Figure S4: Determining the effects of surveillance effort on risk. In (a) we plot the risk as we vary the surveillance effort, $s$, within a 25km CZ. In (b) we plot the risk as we vary the surveillance effort within a 23km BZ which proceeds a 2km perfect EZ. All other parameters and interpretations are as in Fig. S8. Also see Fig. 4 in the main text where we plot the relative changes in risk.

**References**

EFSA PLH Panel (EFSA Panel on Plant Health) (2015) Scientific Opinion on the risks to plant health posed by Xylella fastidiosa in the EU territory, with the identification and evaluation of risk reduction options. EFSA Journal 13:262

European Commission (2014) Final report of an audit carried out in Italy from 10 to 14 February 2014 in order to evaluate the situation and official controls for Xylella fastidiosa. In. http://ec.europa.eu/food/fvo/audit_reports/details.cfm?rep_id=3285#

European Union (2015) Commission Implementing Decision (EU) 2015/789 of 18 May 2015 as regards measures to prevent the introduction into and the spread within the Union of Xylella fastidiosa (Wells et al.) (notified under document C(2015) 3415) In. http://eur-lex.europa.eu/legal-content/EN/TXT/?uri=CELEX:32015D0789

Hirzel AH, Le Lay G, Helfer V, et al. (2006) Evaluating the ability of habitat suitability models to predict species presences. Ecol. Model. 199:142-152

Real R, Barbosa AM, Vargas JM (2006) Obtaining environmental favourability functions from logistic regression. Environ. Ecol. Stat. 13:237-245
